# Genomic differences between nasal *Staphylococcus aureus* from hog slaughterhouse workers and their communities

**DOI:** 10.1371/journal.pone.0193820

**Published:** 2018-03-06

**Authors:** Yaqi You, Li Song, Bareng A. S. Nonyane, Lance B. Price, Ellen K. Silbergeld

**Affiliations:** 1 Department of Environmental Health Sciences, Bloomberg School of Public Health, Johns Hopkins University, Baltimore, Maryland, United States of America; 2 Department of Computer Science, Johns Hopkins University, Baltimore, Maryland, United States of America; 3 Center for Computational Biology, McKusick-Nathans Institute of Genetic Medicine, Johns Hopkins University, Baltimore, Maryland, United States of America; 4 Department of International Health, Bloomberg School of Public Health, Johns Hopkins University, Baltimore, Maryland, United States of America; 5 Department of Environmental and Occupational Health, Milken Institute School of Public Health, George Washington University, Washington, DC, United States of America; 6 Division of Pathogen Genomics, Translational Genomics Research Institute, Flagstaff, Arizona, United States of America; Rockefeller University, UNITED STATES

## Abstract

New human pathogens can emerge from the livestock-human interface and spread into human populations through many pathways including livestock products. Occupational contact with livestock is a risk factor for exposure to those pathogens and may cause further spreading of those pathogens in the community. The current study used whole genome sequencing to explore nasal *Staphylococcus aureus* obtained from hog slaughterhouse workers and their community members, all of whom resided in a livestock-dense region in rural North Carolina. Sequence data were analyzed for lineage distribution, pathogenicity-related genomic features, and mobile genetic elements. We observed evidence of nasal *S*. *aureus* differences between hog workers and non-workers. Nasal *S*. *aureus* from hog workers showed a greater lineage diversity than nasal *S*. *aureus* from community residents. Hog worker isolates were less likely to carry the φSa3 prophage and human-specific immune evasion cluster genes than community resident isolates (φSa3 prophage: 54.5% vs. 91.7%, Benjamini-Hochberg (BH) corrected *p* = 0.035; immune evasion cluster genes: 66.7% vs. 100%, BH *p* = 0.021). Hog worker isolates had a lower prevalence and diversity of enterotoxins than community resident isolates, particularly lacking the enterotoxin gene cluster (39.4% vs. 70.8%, BH *p* = 0.125). Moreover, hog worker isolates harbored more diverse antibiotic resistance genes, with a higher prevalence of carriage of multiple resistance genes, than community resident isolates (75.8% vs. 29.2%, BH *p* = 0.021). Phylogenetic analysis of all ST5 isolates, the most abundant lineage in the collection, further supported separation of isolates from hog workers and non-workers. Together, our observations suggest impact of occupational contact with livestock on nasal *S*. *aureus* colonization and highlight the need for further research on the complex epidemiology of *S*. *aureus* at the livestock-human interface.

## Introduction

*Staphylococcus aureus* is an important pathogen causing significant clinical and public health burdens. This situation is more serious now because of the global emergence of antibiotic resistance, including methicillin resistance, in *S*. *aureus* [1, 2]. In the United States, the Centers for Disease Control and Prevention reported that methicillin resistant *S*. *aureus* (MRSA) was responsible for 11,000 deaths from invasive skin and soft tissue infections in 2011 [3]. Community-associated MRSA (CA-MRSA) now accounts for a significant portion of all MRSA infections, in addition to health care-associated MRSA (HA-MRSA) [3, 4]. Although certain risk factors for infections by CA-MRSA have been characterized [3], reservoirs of resistant and/or virulent *S*. *aureus* outside of health care settings remain poorly understood, and the origins and transmission routes of *S*. *aureus* in the community are not elucidated [5].

The widespread use of antibiotics in industrial food animal production has been a major driver of the development and dissemination of resistance among microbes in agricultural settings and results in human exposure to resistant pathogens via food and the environment [6]. For instance, Rinsky et al. [7] reported that industrial livestock operation workers carried nasal *S*. *aureus* with characteristics that were putatively associated with livestock (e.g., tetracycline-resistant and *scn*-negative), which were not seen in workers from antibiotic-free livestock operations. Recent studies further show that livestock-associated occupational groups are at increased risk of nasal carriage of antibiotic resistant *S*. *aureus*, including MRSA and multidrug resistant *S*. *aureus* (MDRSA), compared to the general population [8–10]. While findings to date indicate that occupational contact with livestock is a risk factor for exposure to livestock-associated *S*. *aureus*, including resistant strains, few studies have examined influences of such exposure on the *S*. *aureus* population in human nostrils, although this information could have important implications for the epidemiology of *S*. *aureus*.

Whole genome sequencing (WGS) and comparative genomics has provided broad insights into the success of *S*. *aureus* as a commensal and pathogen of both humans and animals, revealing genomic features associated with antibiotic resistance, adhesion to and invasion of host cells and tissues, evasion of immune responses, and biofilm formation [2, 11]. Many of these determinants are located on mobile genetic elements (MGEs), such as *S*. *aureus* pathogenicity islands (SaPIs), prophages, and plasmids. Indeed, MGEs account for 15–20% of the *S*. *aureus* genome, accelerating genotypic/phenotypic variation in *S*. *aureus* populations and facilitating the rapid spread of antibiotic resistance and/or virulence through horizontal gene transfer [2, 12]. Comparisons of strains from various host species further suggest a correlation between MGEs acquisition/loss and host adaptation [13–15].

In our previous cross-sectional study conducted at a hog slaughter/processing plant in a livestock-dense region in North Carolina, we found that although the prevalence of nasal carriage of *S*. *aureus* was similar in hog workers and their household and community members, *S*. *aureus* isolates from hog workers were resistant to a greater number of antibiotic classes [9]. Whether that reflected distinct nasal *S*. *aureus* populations in hog workers and non-workers was unclear. The present study sought to use WGS to explore genomic features of nasal *S*. *aureus* collected in our previous study, including isolates colonizing hog workers, their household members, and non-worker residents in the broader community. Our analysis provides new insights into the epidemiology of *S*. *aureus* at the livestock-human interface.

## Materials and methods

### Bacterial isolates

This study included 79 nasal *S*. *aureus* isolates obtained in our previous study [9]. Study design, informed consent, participant enrollment, and biological sampling are detailed therein. Briefly, we enrolled 162 hog slaughterhouse workers, 63 household members of those workers from 50 different households, and 111 community residents, and collected specimens from both nares of each participant using a swab. *S*. *aureus* was isolated and identified from nasal swabs by the Johns Hopkins Hospital Clinical Microbiology Laboratory. One isolate per *S*. *aureus*-positive individual was analyzed in this study (a flowchart in S1 Fig).

We also included 22 previously published sequences of human and livestock strains from international studies for comparison (S1 Table). They represent the same multilocus sequence types (MLSTs) and/or clonal complexes (CCs) as the nasal isolates obtained by us.

### DNA preparation and genome sequencing

Isolates were grown on trypticase soy agar with 5% sheep blood (BBL, BD Diagnostic Systems) at 37°C overnight. DNA was extracted from bacterial cells using a boiling and freeze/thaw method [16]. DNA quantity and quality was assessed using a NanoDrop 2000C spectrophotometer (NanoDrop Technologies).

Genomic libraries were constructed as previously described [14]. Multiplexed paired-end sequencing was conducted at the Institute of Genomic Sciences, University of Maryland Baltimore. Individual libraries were quantified by quantitative PCR on an ABI 7900HT fast real-time PCR system (Life Technologies) before pools of indexed libraries were loaded onto a flow cell for sequencing. Thirty-five isolates were sequenced on the Illumina MiSeq platform (Illumina, Inc.) to a read length of 251 bp and forty-four isolates on the HiSeq 2500 platform (Illumina, Inc.) to a read length of 101 bp. The median approximate coverage was 102 for all the sequenced isolates.

### Quality control and *de novo* assembly

Raw reads were assessed for quality using FASTQC (http://www.bioinformatics.bbsrc.ac.uk/projects/fastqc/) and trimmed at both 5’ and 3’ ends using Trimmomatic [17] to filter out reads of low quality (Phred quality score <30, length <36 bp). Potential contaminating sequences were checked using Kraken [18]; sequencing errors were corrected using Lighter [19]. Corrected reads were *de novo* assembled with SPAdes 3.5.0 [20]; assembly quality was evaluated by QUAST [21]. Contigs of >200 bp and with ≥5× average coverage depth were subject to further analysis.

### MLST and *spa* typing

MLST of most of the isolates has been reported [9]. Several previously undetermined isolates were characterized here using srst2 [22], which employs bowtie2 [23] to call loci directly from Illumina reads based on the *S*. *aureus* MLST database (http://saureus.mlst.net). *spa* types of all the isolates were determined by identifying *spa* repeats (http://spa.ridom.de) in the assemblies using BLAST [24], followed by manual verification.

### Analysis of virulence and antimicrobial resistance genes

For all the isolates, we screened their genome assemblies against the VirulenceFinder database (https://cge.cbs.dtu.dk/services/data.php) [25] and searched for known staphylococcal enterotoxins (SEs) (*sea*–*see*, *seg*–*seq* and *yent2*) as described in [26]. The presence of immune evasion cluster (IEC) genes, including *chp* for chemotaxis inhibitory protein, *scn* for staphylococcal complement inhibitor, *sea* or *sep* for staphylococcal enterotoxin A or P, and *sak* for staphylokinase, was examined using a method similar to Price et al. [14]. S2 Table lists all virulence factors analyzed in this study.

To identify antibiotic resistance genes, we integrated three databases, namely ARG-Annot [27], ResFinder [28], and CARD [29], which was used to interrogate the isolate genome assemblies.

*S*. *aureus* frequently contains genes conferring resistance to heavy metals, antiseptics and disinfectants that co-exist with virulence and/or antibiotic resistance genes on MGEs [30]. Here we examined a set of those genes known to be encoded by MGEs, including arsenic resistance genes *arsA*, *arsB*, *arsC*, *arsD* (accession numbers AB505628, AB505628, AB505628, AB505628), cadmium resistance genes *cadD* and *cadX* (AP003139), and antiseptic resistance genes *qacA/B* and *qacC* (CP012121, NC_005054).

All genes were identified with a threshold of ≥90% nucleotide identity, ≥90% length coverage, and ≥5× sequence depth.

### Identification of MGEs

We focused on three types of MGEs that are often associated with enhanced *S*. *aureus* pathogenicity: SaPIs, prophages, and plasmids. SaPIs were identified by their integrase (*int*) genes based on the current classification, which includes SaPI1028, SaPI1, SaPI2, SaPI3, SaPI4, SaPI5, SaPI6Δ, SaPIbov1, SaPIbov2, SaPIbov3, SaPIbov4, SaPIfusB, SaPIm1, SaPIm4, SaPImw2, and SaPIn1 [31, 32] (S3 Table). The presence of two prophages, φSa2 and φSa3, was assessed by their *int* genes as described in [14]. The φSa2 prophage encodes Panton-Valentine leucocidin (PVL), a key factor in hemolytic pneumonia and skin and soft tissue infection and epidemiologically associated with CA-MRSA [4, 33]. The φSa3 prophage harbors IEC genes that allow *S*. *aureus* to compromise the effectiveness of neutrophils and macrophages [34, 35]. Types of φSa3 were classified according to van Wamel et al. [36]. To identify plasmids, the genome assemblies were screened against the PlasmidFinder database (https://cge.cbs.dtu.dk/services/data.php) for plasmid replicon (*rep*) genes [37]. Occasionally, staphylococcal chromosomal cassette *mec* elements (SCC*mec*) were identified in certain isolates, and their types were classified accordingly [38].

### Phylogenetic analysis and genome annotation for the ST5 lineage

Sequence type (ST) 5 was the most abundant lineage in this study (n = 19) and was found in all population groups, providing an opportunity for a deeper analysis. Consequently, a whole genome phylogeny was reconstructed for this lineage using REALPHY [39]. Briefly, the 19 ST5 genome assemblies from this study, along with 6 published ST5 genomes, were mapped to 2 ST105 reference genomes by bowtie2 [23]. ST105 was chosen as reference because of its relatedness to ST5 within CC5 [26]. The resulting alignments were merged, and single nucleotide polymorphisms (SNPs) and nonpolymorphic positions were used for generating a maximum likelihood phylogeny using PhyML with the generalized time-reversible substitution model, the gamma-distributed rate heterogeneity, and midpoint rooting [39, 40].

Genome assemblies of the 19 ST5 isolates were further annotated in the CloVR-Microbe pipeline [41]. Identified SaPIs and plasmids were compared to SaPI and *S*. *aureus* plasmid sequences in the GenBank database using Mauve [42]. In addition, the 19 annotated ST5 genome assemblies were input to the CloVR-Comparative pipeline, which uses reference-free whole genome alignment to call SNPs for phylogeny reconstruction [43].

### Statistical analysis

All statistical analyses were performed using R (version 3.2.4). Simpson diversity and 95% confidence interval were calculated as described previously [44]. The presence/absence of individual genes was compared between isolates from different population groups, using community resident isolates as reference. Differences between proportions of gene-carriage were tested by two-sided Fisher-Boschloo exact test implemented in the “Exact” package [45], and *p*-Value correction for multiple testing was conducted using the Benjamini-Hochberg (BH) method [46] implemented in the “stats” package. Difference between mean numbers of SEs or antibiotic resistance genes per genome was tested by two-sided ANOVA or Welch *t* test after Levene’s test for variance homogeneity.

### Nucleotide accession numbers

The Illumina sequences generated and used in this study are deposited in the NCBI Sequence Read Archive (SRA) under the BioProject accession number PRJNA412599.

## Results

### Lineage diversity of nasal *S*. *aureus* in a community of hog slaughterhouse workers and residents in a region with intensive swine production

In this study, we successfully sequenced 77 of the 79 nasal isolates that were obtained from 336 residents in rural North Carolina (WGS statistics in S4 Table). The MLST of one isolate was untypable and that isolate was excluded from further analysis, leaving a total of 76 sequenced isolates for genomic analysis. These isolates belonged to 26 STs, including two different single-locus variants (SLVs) of ST8, one SLV of ST15, one SLV of ST30, one SLV of ST45, one SLV of ST630, and one novel ST. All the SLVs had one SNP difference from the closest STs (S2 Fig). Among the identified STs, ST5 was most abundant (19/76; 25%), followed by ST8 and SLVs of ST8 (12/76; 16%), ST1 (7/76; 9%), and ST45 and SLVs of ST45 (7/76; 9%) (S5 Table). Except for one ST45 isolate and one isolate with a novel ST, *spa* types were determined for these isolates. A total of 46 *spa* types were identified, including 5 novel types. For many STs in this study, multiple *spa* types were observed (S2 Fig and S5 Table).

### Virulence genes in nasal *S*. *aureus*

**Fig 1:**
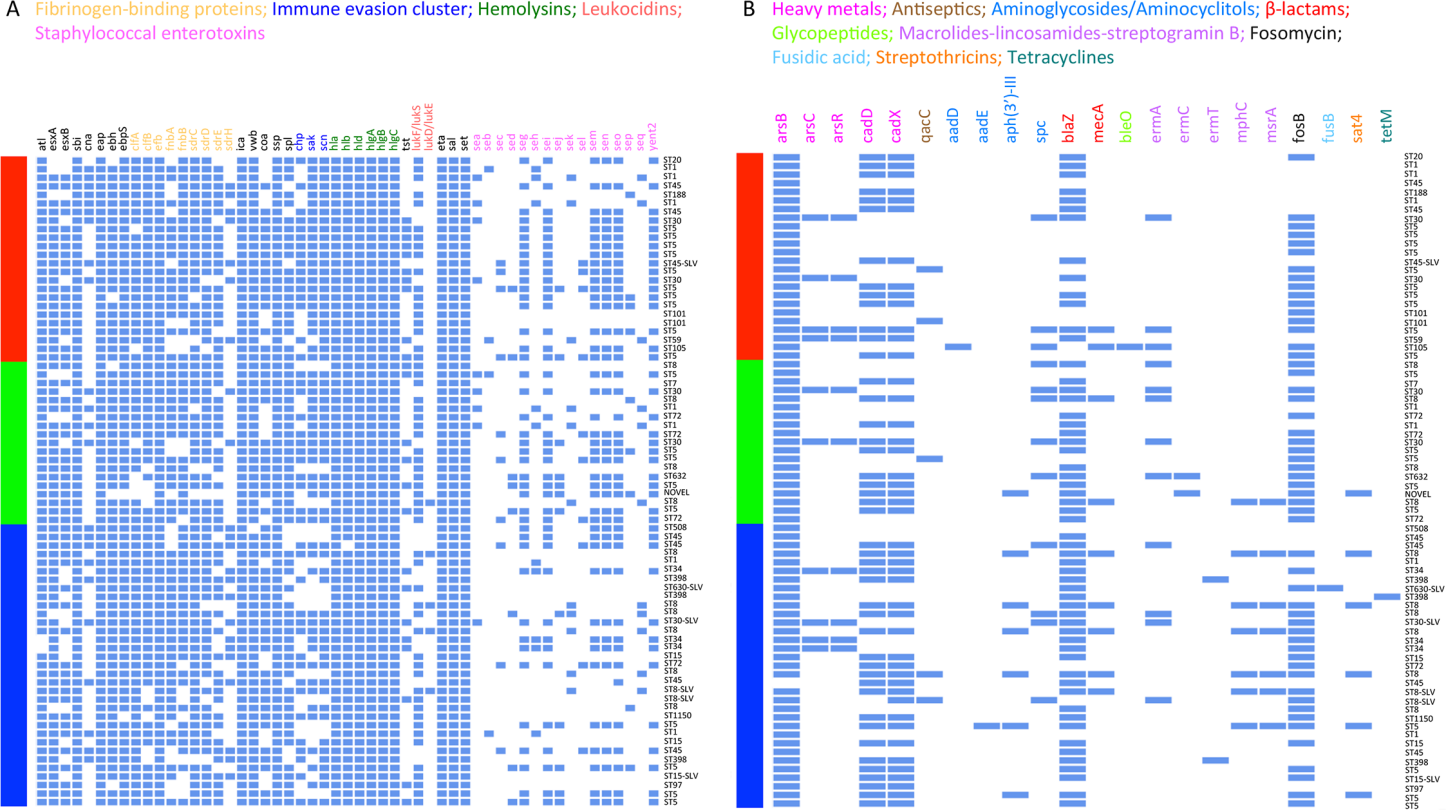
**Profiles of (A) virulence factors and (B) antimicrobial resistance genes for the 76 sequenced nasal *S*. *aureus* isolates.** Each row represents one isolate from an individual, with the color bar on the left side indicating host groups (red, community residents; green, household members; blue, hog workers) and the texts on the right side indicating lineages.

A variety of virulence genes were identified in the 76 isolates (Fig 1A and S5 Table). They encode adhesins, exoenzymes, toxins and other virulence factors responsible for adhesion to and invasion of host cells and tissues, evasion of immune responses, and biofilm formation (S2 Table).

All isolates contained genes that encode fibrinogen-binding proteins (*clfA*, *clfB*, *efb*, *fnbA*, *fnbB*, *sdrC sdrD*, *sdrE*, *sdrH*), with *efb* being the most prevalent (76/76;100%) and *sdrH* being the least prevalent (23/76; 30%). Other adhesin genes, including *eap*, *ebh*, *ebpS*, *ica* (*icaA*, *icaB*, *icaC*, *icaD*, *icaR*) and *vwb*, were also common (93–100%), but the collagen adhesin gene *cna* was relatively less prevalent (23/76; 30%).

Genes encoding exoenzymes, such as *coa* for Staphylocoagulase, *sspB* for cysteine protease, and *slp* (*splA*, *splB*, *splC*, *splD*, *splF*) for serine protease-like proteins, were present in most isolates (72%-100%). Many isolates had at least one IEC gene (62/76; 82%), with *scn* being the most prevalent (62/76; 82%), followed by *sak* (56/76; 74%), and then *chp* (44/76; 58%).

Diverse toxins were identified in these isolates. Hemolysin genes were present in all isolates, with 75 isolates simultaneously containing *hla*, *hlb*, *hld*, *hlgA*, *hlgB* and *hlgC* and one ST45 isolate containing *hla*, *hld*, *hlgA*, *hlgB* and *hlgC*. A small portion of these isolates had the *tst* gene encoding toxic shock syndrome toxin-1 (24/76; 32%). The *lukF*-*PV* and *lukS-PV* genes encoding PVL were rare, only present in four ST8 isolates and one ST8-SLV isolate (5/76; 7%). The leukocidin-encoding genes *lukD* and *lukE* were more common (56/76; 74%). The exfoliative toxin gene *eta*, as well as superantigen-like genes *sal* and *set*, were present in all isolates (76/76; 100%).

Except for the *see* gene, other known SEs (*sea*–*sed*, *seg*–*seq* and *yent2*) were identified among these isolates. Individual isolates had varying SE profiles. Sixteen isolates did not contain any SE (16/76; 21%), while two ST5-t002 isolates each contained 10 SEs. Forty-one isolates had the *seg*, *sei*, *sem*, *sen* and *seo* genes of the enterotoxin gene cluster (*egc*) (41/76; 54%). Forty-two isolates (42/76; 55%) contained the non-functional pseudogene *yent2* that is also located in the *egc* oepron and can rearrange with another pseudogene *yent1* to yield the functional enterotoxin gene *seu*.

### Antimicrobial and heavy metal resistance genes in nasal *S*. *aureus*

A total of 16 antibiotic resistance genes were identified in the 76 isolates (Fig 1B and S5 Table). They encode resistance to macrolides-lincosamides-streptogramin B antibiotics (MLS_B_) (*ermA*, *ermC*, *ermT*, *mphC*, *msrA*), aminoglycosides/aminocyclitols (*aadD*, *aadE*, *aph(3’)-III*, *spc*), β-lactams (*blaZ*, *mecA*), glycopeptides (*bleO*), fosfomycin (*fosB*), fusidic acid (*fusB*), streptothricins (*sat4*), and tetracyclines (*tetM*) (S6 Table). Among these identified genes, the penicillin resistance gene *blaZ* (57/76; 75%) and the fosfomycin resistance gene *fosB* (54/76; 71%) were the most prevalent, followed by *spc* encoding spectinomycin resistance (12/76; 16%) and *ermA* conferring MLS_B_ resistance (12/76; 16%). Resistance gene profiles varied for individual isolates. For example, one ST5 isolate contained 7 resistance genes, while five isolates of ST1, ST45, ST97 and ST508 did not have any resistance gene.

Among the genes for heavy metal and antiseptic resistance that were examined in this study, the arsenic resistance gene *arsB* was the most prevalent (75/76; 99%), followed by the cadmium resistance genes *cadD* (45/76; 59%) and *cadX* (46/76; 61%). The other arsenic resistance genes (*arsC*, *arsR*) were less common (10/76; 13% for each gene), and the quaternary ammonium compound resistance gene *qacC* was rare (5/76; 7%) (Fig 1B and S5 Table).

### Carriage of MGEs among nasal *S*. *aureus*

Analysis of the 76 isolate genomes identified diverse MGEs, including SaPIs, prophages, plasmids, and SCC*mec* elements (S5 Table). SaPIs were found in many isolates (50/76; 66%). Isolates of certain lineages, such as ST15 and ST34, showed consistent SaPI *int* profiles. But for many lineages (e.g., ST1, ST5, ST8 and ST8-SLVs, ST30 and ST30-SLV, ST45 and ST45-SLV, ST72), varying SaPI *int* profiles were observed in individual isolates. Specifically, SaPI6Δ (i.e., νSa4 type II) was the most prevalent class (21/76; 28%), present in isolates of ST1, ST8 and ST8-SLVs, ST1150, and a novel ST.

The φSa2 prophage was present in certain isolates (19/76; 25%). A small portion of the φSa2-positive isolates also carried PVL genes (5/76; 7%). φSa3 prophages were common among isolates (56/76; 74%), and they harbored IEC genes in various combinations. A small number of the φSa3 prophages also harbored one enterotoxin gene, either *sea* or *sep* (9/76; 12% for each gene).

Plasmids were identified in many isolates (64/76; 84%). For certain lineages (e.g., ST30 and ST30-SLV, ST34), the same plasmid *int* profiles were seen in individual isolates. However, many lineages (e.g., ST1, ST5, ST8 and ST8-SLVs, ST15 and ST15-SLV, ST45 and ST45-SLV, ST72) showed varying plasmid *int* profiles across individual isolates.

SCC*mec* elements were identified in 9 isolates (9/76; 12%) (7 isolates of ST8 and ST8-SLV, 1 ST5 isolate, 1 ST105 isolate). The majority of these isolates contained SCC*mec* IV (8/9) while one ST105 isolate contained SCC*mec* II.

### Nasal *S*. *aureus* of the ST5 lineage

**Fig 2:**
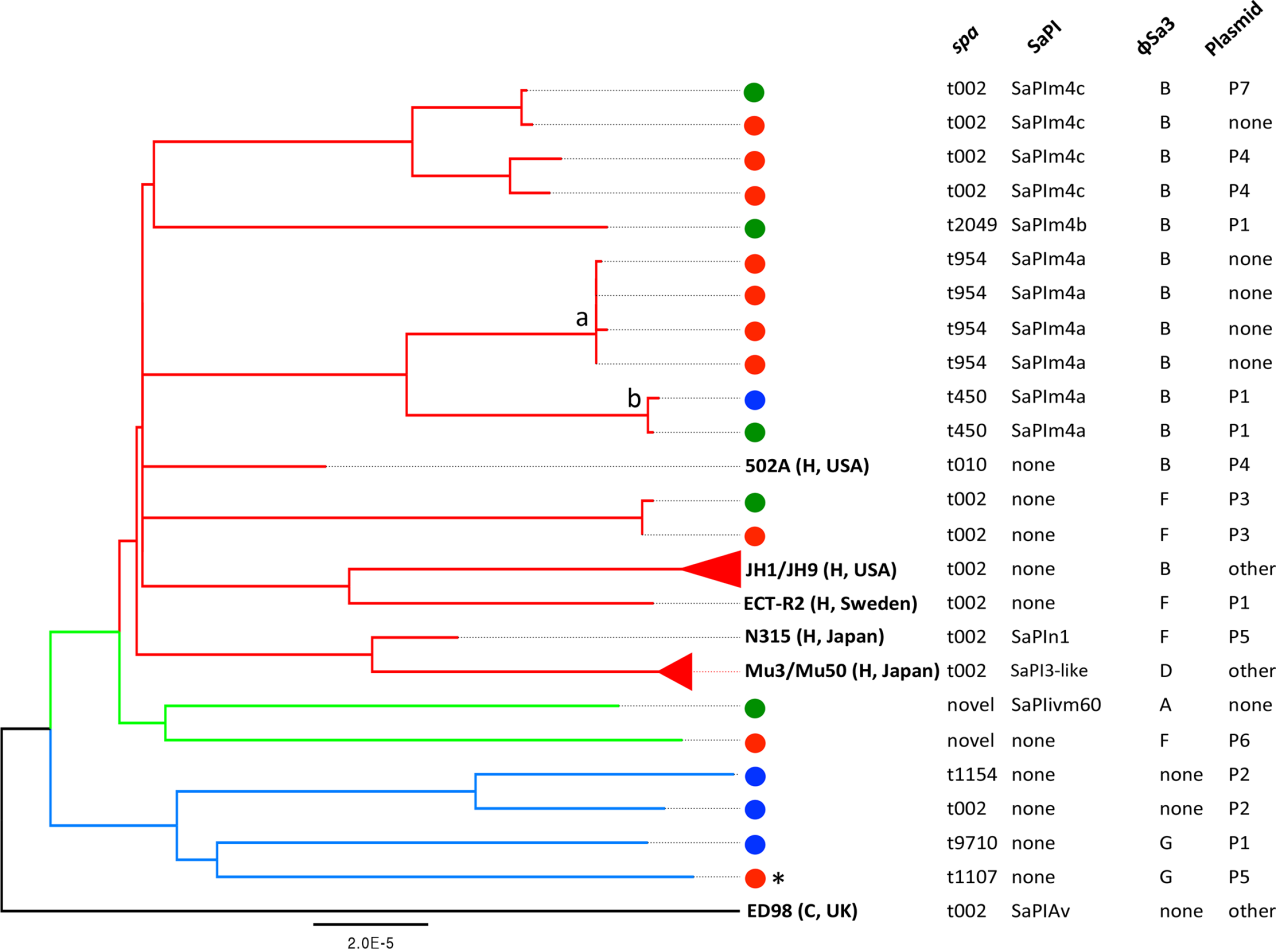
Maximum likelihood phylogeny of the 19 ST5 *S*. *aureus* isolates sequenced in this study. Published genome sequences of 6 ST5 strains (502A, ECT-R2, ED98, Mu3, Mu50, N315) and 2 ST105 strains (JH1 and JH9) were included for comparison, with the country of report and host (H, human; C, chicken) for each reference strain shown in parenthesis (details in S1 Table). Color dots represent host groups in this study (red, community residents; green, household members; blue, hog workers). *spa* types and MGE types are listed on the right of the phylogeny. Subgroups a and b include isolates of certain relatedness, with subgroup b coming from the same household. Asterisk denotes a ST5 MRSA (SCC*mec* IV) isolate.

A whole-genome phylogeny was constructed for ST5, the most abundant lineage in the collection (Fig 2 and S3 Fig). The 19 nasal isolates clustered into two major clades. Clade I (red and green branches) included most isolates from community residents (9/10) and all isolates from household members of hog workers (5/5), as well as the CC5 reference strains from human (502A, ECT-R2, JH1, JH9, Mu3, Mu50, N315). Clade II (blue branch) included most isolates from hog workers (3/4) and one community resident isolate (1/10). Within Clade I, several isolates showed certain relatedness, including four community resident isolates (subgroup a) and two isolates from a hog worker’s household (subgroup b).

A variety of MGEs were present in these ST5 isolates (Fig 2). Two classes of SaPIs were present in 12 of the 19 nasal isolates. The SaPIm4 (i.e., νSa3 type I) class was predominant, present in most community resident isolates (7/10) and household member isolates (3/5), as well as in one hog worker isolate (1/4). These SaPIm4 elements fell into three subtypes (SaPIm4a, SaPIm4b, SaPIm4c), which shared 98–99% nucleotide identity over a large region but had various genes adjacent to their *attR* sites (S4A Fig). In detail, SaPIm4a carried a *fhuD* gene encoding ferrichrome-binding protein and a *lcm* gene encoding leucine carboxyl methyltransferase. SaPIm4b carried an *ear* gene encoding extracellular β-lactamase homolog, in addition to *fhuD* and *lcm*. SaPIm4c carried *ear* and two enterotoxin genes, *sec* and *sel*. The second SaPI class, an element akin to SaPI3 (i.e., νSa1), was present in a household member isolate (1/5). It had *ear* and the enterotoxin gene *seb* adjacent to its *attR* (S4A Fig). Overall, SaPIs in these nasal isolates seemed to have evolved from recombination between SaPIs of various *S*. *aureus* strains reported around the world [47–49].

Besides SaPIs, seventeen of the 19 nasal isolates carried φSa3 prophages. The two isolates without the φSa3 prophage were both from hog workers. Among the 17 φSa3-bearing isolates, most had type B φSa3 (11/17) and others had φSa3 of type A (1/17), type F (3/17), and type G (2/17) (Fig 2). Notably, all the reference strains from clinical settings (502A, JH1, JH9, ECT-R2, N315, Mu3, Mu50) contained φSa3 prophages.

Furthermore, thirteen of the 19 nasal isolates had ≥20 kb plasmids, including half of the community resident isolates (5/10), most household member isolates (4/5), and all of the hog worker isolates (4/4) (Fig 2). These plasmids (P1–P7) were similar to those previously found in various *S*. *aureus* strains, carrying accessary genes encoding penicillinase, enterotoxins, efflux transporters for quaternary ammonium compounds, cadmium transporters, and bacteriocin ABC transporters (S4B Fig).

### Nasal *S*. *aureus* from hog workers, their household members and community residents

During analysis of the 76 nasal isolates, several trends were noted. Overall, isolates from hog slaughterhouse workers showed greater lineage diversity than isolates from non-worker groups (S5 Fig). A total of 16 STs/SLVs and 26 *spa* types were seen among hog worker isolates (n = 33), while only 8 STs/SLVs and 14 *spa* types were observed among isolates from household members of hog workers (n = 19), and 10 STs/SLVs and 12 *spa* types were seen among isolates from community residents (n = 24). Based on MLST types, Simpson diversity index was 0.934 (95% CI: 0.897−0.971) for nasal isolates from hog workers, 0.877 (95% CI: 0.808−0.947) for nasal isolates from worker’s household members, and 0.815 (95% CI: 0.677−0.953) for nasal isolates from community residents. Based on *spa* types, Simpson index was 0.976 (95% CI: 0.944−1.008) for hog worker isolates, 0.967 (95% CI: 0.927−1.007) for household member isolates, and 0.920 (95% CI: 0.870−0.971) for community resident isolates.

Furthermore, there was evidence suggesting distinct genomic features of nasal *S*. *aureus* from different population groups. In general, nasal *S*. *aureus* from hog workers was less likely to contain any human-specific IEC gene than isolates from community residents (66.7% vs. 100%, BH corrected *p* = 0.021) (Table 1). The prevalence of the staphylokinase-encoding gene (*sak*) and the staphylococcal complement inhibitor gene (*scn*) was significantly lower among hog worker isolates than community resident isolates (51.5% vs. 95.8%, BH *p* = 0.012 for *sak*; 66.7% vs. 100%, BH *p* = 0.021 for *scn*). The prevalence of the chemotaxis inhibitory protein-encoding gene (*chp*) was also lower in hog worker isolates than in community resident isolates (48.5% vs. 66.7%), although not statistically significant. Nasal *S*. *aureus* from household members of hog workers and community residents did not show statistical differences in carriage of IEC genes (Table 1).

**Table 1:** Characteristics of nasal *S*. *aureus* from hog slaughterhouse workers, their household members, and community residents. Percentages indicate prevalence among isolates analyzed from participants of each population group. *p*_*1*_: Comparisons between hog worker isolates and community resident isolates. *p*_*2*_: Comparisons between household member isolates and community resident isolates.

	Hog worker^*a*^ (n = 33)	Household member (n = 19)	Community resident (n = 24)	*p*-Value^*b*^		BH-adjusted *p*-Value^*b*^	
				*p*_*1*_	*p*_*2*_	*p*_*1*_	*p*_*2*_
**MLST type^*c*^**	16 types	8 types (1 novel)	10 types	−	−	−	−
***spa* type**	26 types (3 novel)^*d*^	14 types (1 novel)^*e*^	12 types (1 novel)^*f*^	−	−	−	−
**Plasmid**	29 (87.9%)	18 (94.7%)	17 (70.8%)	0.145	**0.042**	0.440	0.648
**SaPI**	21 (63.6%)	15 (78.9%)	14 (58.3%)	0.760	0.162	0.979	0.913
**φSa2**	10 (30.3%)	4 (21.1%)	5 (20.8%)	0.488	1.000	0.752	1.000
**φSa3**	18 (54.5%)	16 (84.2%)	22 (91.7%)	**0.002**	0.541	**0.035**	1.000
***scn***	22 (66.7%)	16 (84.2%)	24 (100%)	**<0.001**	0.065	**0.021**	0.795
***sak***	17 (51.5%)	16 (84.2%)	23 (95.8%)	**<0.001**	0.241	**0.012**	0.913
***chp***	16 (48.5%)	12 (63.2%)	16 (66.7%)	0.181	1.000	0.513	1.000
**SEs^*g*^**	22 (66.7%)	16 (84.2%)	22 (91.7%)	**0.023**	0.541	0.125	1.000
***egc*^*h*^**	13 (39.4%)	11 (57.9%)	17 (70.8%)	**0.023**	0.456	0.125	1.000
***yent2***	13 (39.4%)	12 (63.2%)	17 (70.8%)	**0.023**	0.676	0.125	1.000
***sea***	1 (3.0%)	4 (21.1%)	4 (16.7%)	**−**	1.000	−	1.000
***sep***	2 (6.1%)	3 (15.8%)	4 (16.7%)	0.184	1.000	0.513	1.000
***clfB***	28 (84.8%)	15 (78.9%)	24 (100%)	0.060	**0.021**	0.224	0.629
***tst***	8 (24.2%)	6 (31.6%)	10 (41.7%)	0.198	0.496	0.532	1.000
**PVL**	4 (12.1%)	1 (5.3%)	0	**−**	**−**	−	−
***lukE-lukD***	21 (63.6%)	17 (89.5%)	18 (75.0%)	0.350	0.221	0.735	0.913
***Multi-resistance gene*^*i*^**	25 (75. 8%)	14 (73.7%)	7 (29.2%)	**<0.001**	**0.004**	**0.021**	0.243
***blaZ***	28 (84.8%)	16 (84.2%)	13 (54.2%)	**0.012**	**0.035**	0.125	0.648
***aph*(*3’*)-*III***	6 (18.2%)	1 (5.3%)	0	−	−	**−**	−
***mphC***	6 (18.2%)	1 (5.3%)	0	−	−	−	−
***msrA***	6 (18.2%)	1 (5.3%)	0	−	−	−	−
***sat4***	5 (15.2%)	1 (5.3%)	0	−	−	−	−

^*a*^n, number of *S*. *aureus* isolates. One isolate per *S*. *aureus*-positive participant was sequenced. See S1 Fig for number of persons enrolled and tested for nasal *S*. *aureus* in the parent cross-sectional study

^*b*^*p*-Values were calculated in Fisher-Boschloo exact test, not available for some characteristics due to data type or small sample size; *p*-Values were corrected for multiple testing with the Benjamini–Hochberg method

^*c*^Including five SLVs that each had one SNP from the closest ST. SLVs were considered as individual types

^*d*^The *spa* type of one ST45 isolate was untypable. Three novel *spa* types were 04-522-12-21-17-34-22-25 for a ST630-SLV isolate, 04-34-24-34-22-24-34-22-25 for a ST8 isolate, and 07-23-12-34-12-10-23-02-12-23 for a ST15

^*e*^The *spa* type of one isolate with a novel ST was untypable. One novel *spa* type was 26-23-17-34-17-20-17-12-17-17-17-16-16 for a ST5 isolate

^*f*^One novel *spa* type was 16-23-22-17-12-17-16 for a ST5 isolate

^*g*^Containing at least one of the known SEs (*sea*–*see*, *seg*–*seq* and *yent2*)

^*h*^Containing five SEs of the enterotoxin gene cluster (*seg*, *sei*, *sem*, *sen* and *seo*).

^*i*^Containing at least two antibiotic resistance genes.

**Fig 3:**
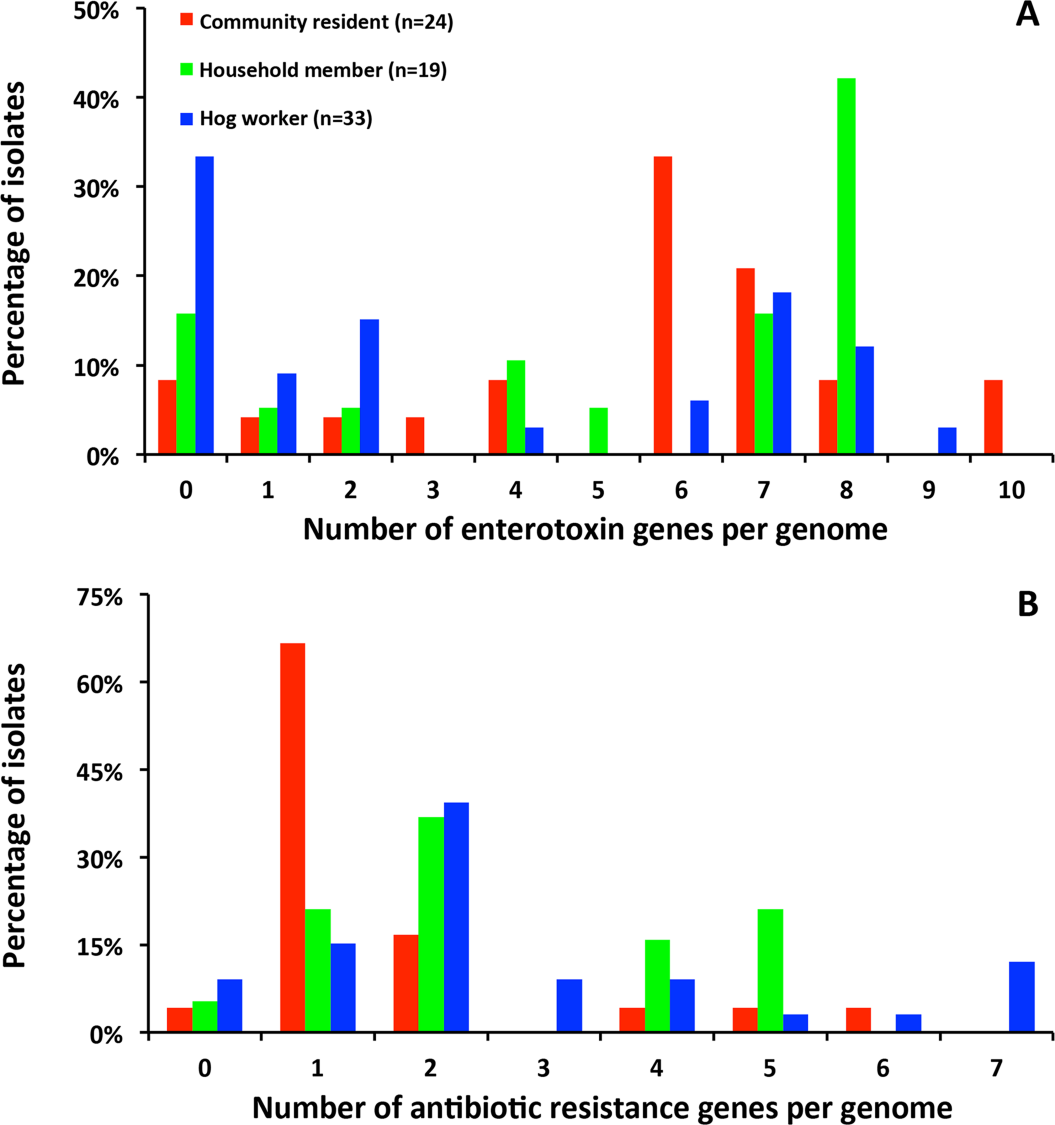
Numbers of (A) staphylococcal enterotoxins and (B) antibiotic resistance genes in the genomes of the nasal *S*. *aureus* of hog workers, their household members, and community residents, respectively.

Nasal *S*. *aureus* from hog workers was also less likely to contain SEs than isolates from community residents (66.7% vs. 91.7%, BH *p* = 0.125) (Table 1). Most hog worker isolates had ≤2 enterotoxins in their genomes, whereas the majority of community resident isolates had ≥5 enterotoxins in their genomes (Fig 3A). Such a discrepancy was largely due to carriage of the *egc* (i.e., *seg*, *sei*, *sem*, *sen*, *seo*, *yent2*), which showed lower prevalence in hog worker isolates as compared to community resident isolates (39.4% vs. 70.8%, BH *p* = 0.125). Nasal *S*. *aureus* from household members of hog workers and community residents were similar in SE carriage (Table 1 and Fig 3A).

Distinct genomic features of nasal *S*. *aureus* from different population groups were also illustrated by antibiotic resistance genes (Table 1). Overall, a total of 13, 10, and 7 classes of resistance genes were observed in isolates from hog workers, their household members, and community residents, respectively. Isolates from hog workers and their household members tended to harbor a greater number of antibiotic resistance genes in their genomes than isolates from community residents (mean number being 2.8, 2.6 and 1.6; *p* = 0.025 for hog worker isolates vs. community resident isolates; *p* = 0.036 for household member isolates vs. community resident isolates) (Fig 3B). The prevalence of carrying ≥2 resistance genes was significantly higher among isolates from hog workers than those from community residents (75.8% vs. 29.2%, BH *p* = 0.021). Specifically, isolates from hog workers were more likely to contain the penicillin resistance gene *blaZ* (84.8% vs. 54.2%, BH *p* = 0.125). Isolates from household members also showed higher prevalence of carrying ≥2 resistance genes than community resident isolates (84.2% vs. 54.2%), although not statistically significant. Additionally, the aminoglycoside phosphorylase gene *aph*(*3’*)*-III*, two macrolide resistance genes (*mphC* and *msrA*), and the streptothricin acetyltransferase gene *sat4* were present in several hog worker isolates and one household member isolate but were not seen in community resident isolates.

Nasal *S*. *aureus* from the three population groups further showed differences in carriage of prophages and plasmids (Table 1). The prevalence of φSa3 was much lower among hog worker isolates than community resident isolates (54.5% vs. 91.7%, BH *p* = 0.035), while household member isolates and community resident isolates were similar in φSa3 carriage. The prevalence of plasmids was somewhat higher in hog worker isolates and household member isolates than in community resident isolates (87.9%, 94.7% and 70.8% for isolates from workers, household members and community residents, respectively), although not statistically significant.

## Discussion

The growing recognition of *S*. *aureus* as a community-based pathogen urges a comprehensive understanding of reservoirs of *S*. *aureus* outside of health care settings and the origins and transmission routes of *S*. *aureus* in the community. The epidemiology of *S*. *aureus* is complex at the livestock-human interface, where farm animals provide additional reservoirs of *S*. *aureus*. Extensive contact with those animals could lead to human exposure to animal-associated microbiota, which might eventually spread into the community. Bacterial WGS analysis has been used to successfully resolve the evolutionary trajectories of several CA-MRSA strains and in some cases revealed their emergence from the livestock-human interface [15, 50, 51]. Here we applied WGS to analyze nasal isolates that were collected in our previous cross-sectional study conducted at a hog slaughter/processing plant in rural North Carolina. We observed differences in nasal *S*. *aureus*, including lineage diversity and pathogenicity-related genomic features, between hog workers and their community members.

We previously reported greater MLST diversity of nasal isolates from hog workers as compared to those from their household and community members [9]. The current study confirms and extends that observation. Analysis based on both MLST and *spa* typing revealed highest lineage diversity of nasal *S*. *aureus* from hog workers and lowest diversity of nasal *S*. *aureus* from community residents. The exact sources of *S*. *aureus* colonization in the study population are unknown. However, given that all the three population groups were from the same broader community, occupational exposure most likely contributed to the *S*. *aureus* diversity in hog workers. Further clues might come from sampling the animals entering the plant and the working environment inside the plant.

Several lines of evidence suggest distinct genomic features of nasal *S*. *aureus* of livestock-associated workers and non-workers. Compared to community resident isolates, hog worker isolates were less likely to contain φSa3 prophages and φSa3-associated human-specific IEC genes, particularly the *sak* and *scn* genes. φSa3 prophages are present in diverse *S*. *aureus* lineages and are more common in colonizing strains [52]. The associated IEC genes are suggested to facilitate *S*. *aureus* nasal colonization, playing crucial roles in its adaptation to human [34, 53]. Consequently, the relatively high prevalence of φSa3 and human-specific IEC genes in nasal *S*. *aureus* from non-workers could be normal. However, the significantly lower prevalence of φSa3 and human-specific IEC genes in nasal *S*. *aureus* from hog workers was unexpected. It is reported that regardless of *S*. *aureus* strain lineage and geographic location, φSa3 and IEC genes are present in 83%−100% of human isolates but only in 2%−34% of animal isolates, a phenomenon likely resulting from host adaptation [14, 15, 36, 53, 54]. More recently, a study focusing on ST5 MRSA reported the complete absence of φSa3 and IEC genes in all isolates sampled from swine, swine facility environments, and humans with short- or long-term exposure to swine. In contrast, the authors found that 90.4% of the examined clinical isolates from persons with no exposure to swine carried φSa3 prophages with IEC genes [55]. Therefore, the lack of φSa3 and IEC genes in hog worker isolates in this study could suggest a contribution of zoonotic sources to *S*. *aureus* colonization in this occupational group.

Compared to nasal *S*. *aureus* from community residents, hog worker isolates also showed a lower prevalence and less diversity of enterotoxins, particularly lacking the *egc* operon [56]. *egc* was found common in nasal isolates [57] and is suggested to primarily function as a colonization factor, increasing commensal fitness [58]. This may explain its relatively high prevalence in nasal *S*. *aureus* from non-workers. But the lower prevalence of *egc*, as well as all SEs in general, among hog worker isolates was unexpected. Previous analysis of global *S*. *aureus* strains showed a lower carriage of SEs and/or *egc* in animal-associated strains than in human strains [59, 60]. A recent study sampling swine herds from 11 states of the United States, including North Carolina, also found a uniform absence of *sea*–*see* enterotoxins in representative swine isolates, regardless of their MLST backgrounds [61]. Therefore, the observation of less SEs in hog worker isolates in this study supports our hypothesis that zoonotic sources contributed to *S*. *aureus* colonization in this occupational group.

Moreover, nasal *S*. *aureus* from hog workers showed greater diversity of antibiotic resistance genes and significantly higher prevalence of carriage of multiple resistance genes than community resident isolates. This finding is consistent with and extends our previous phenotypic observation [9]. Genes conferring resistance to aminoglycosides/aminocyclitols and MLS_B_ antibiotics were particularly abundant in hog worker isolates. For instance, one hog worker isolate simultaneously carried the *aadE*-*sat4*-*aph*(*3’*)*-III* cluster encoding aminoglycoside and streptothricin modifying enzymes as well as the *mphC*-*msrA* cluster for resistance to macrolides. Another three hog worker isolates simultaneously had the *sat4*-*aph*(*3’*)*-III* cluster and the *mphC*-*msrA* cluster. The accumulation of multiple resistance genes for the same class of drugs in a bacterial genome could result from horizontal gene transfer followed by recombination, which probably reflects certain selective pressure during the strain’s evolution [62]. Drugs including MLS_B_ antibiotics are currently used in swine production and might have direct or indirect influence on the accumulation of resistance genes in hog worker isolates [6]. This is supported by previous reports showing enrichment of clusters of multiple antibiotic resistance genes on MGEs in swine and swine farm environmental microbiomes [63, 64]. Those studies found enrichment of aminoglycoside resistance genes clustered with macrolide resistance genes on MGEs although aminoglycosides were not fed to swine, and suggested coselection as a potential mechanism. Surprisingly, nasal *S*. *aureus* from household members of hog workers also showed greater diversity of antibiotic resistance genes and higher prevalence of carriage of multiple resistance genes than community resident isolates. This raises concerns about potential flow of resistance genes within households of hog workers. Notably, tetracycline resistance was uncommon among our isolates, in contrast to findings of other studies of populations involved in swine farming [7, 8]. This is largely due to the fact that most tetracycline resistant isolates in those studies were ST398 strains, a lineage uncommon in our collection.

Analysis of all ST5 isolates in our collection further indicated differences between nasal *S*. *aureus* of hog workers and non-workers. In the whole-genome inferred phylogeny, isolates from hog workers and non-workers are largely separated. Moreover, hog worker isolates all contained plasmids encoding virulence and penicillin resistance but mostly lacked SaPIs, and only half contained φSa3 prophages. In comparison, the majority of non-worker isolates contained SaPIs with virulence genes and φSa3 prophages with human-specific IEC genes, but many of them lacked plasmids. The ST5 lineage is comprised of both animal and human strains, and MGEs have contributed to the success of globally distributed ST5 human strains and the pathogenicity of frequently emerged ST5 CA-MRSA [13, 31, 65]. Based on swine herds from major swine producing regions in 11 states, ST5 also represents one dominant lineage in swine in the United States [61], and the selected swine-associated ST5 MRSA isolates showed *in vitro* adherence to human keratinocytes equivalent to clinical ST5 MRSA isolates, suggesting humans in contact with swine have the potential to be colonized with those isolates [66]. Our observations, based on WGS analysis, could suggest distinct origins of this lineage in hog workers and non-workers in North Carolina and warrants further research on its dynamics at the livestock-human interface.

Several limitations need to be considered, which might affect this study’s robustness. Only one isolate from each *S*. *aureus*-positive participant was analyzed and for 18 STs/SLVs, our collection only contained 1 to 2 isolates. WGS of multiple isolates per individual has unveiled considerable within-host diversity of *S*. *aureus*, emphasizing the need for analyzing several isolates per person to capture an accurate picture of *S*. *aureus* colonization [67]. Analysis of multiple isolates from each individual could also help explain the sporadic occurrence of certain lineages and genetic variations across lineages [68].

We were not provided access to hogs or the environment of the slaughter/processing plant. This prevented us from confirming that the nasal isolates from hog workers reflected the *S*. *aureus* populations in swine. Further, the hog slaughter/processing plant was located in a livestock-dense area. Hence, the study population may have been exposed to livestock-associated microbiota through environmental pathways.

Overall, however, our data support a growing concern about impacts of occupational contact with livestock on *S*. *aureus* populations in human. Findings from this study also add to our understanding of the epidemiology of *S*. *aureus* in the community, particularly situations in intensive livestock production areas. Further research is needed to confirm findings in this study and to fully understand the origins and transmission routes of *S*. *aureus* in the community.

## Supporting information

S1 Fig Flowchart of isolate collection in the parent cross-sectional study and bacterial WGS in this study. After WGS, three isolates were excluded due to low sequencing quality (n = 2) or untypable MLST (n = 1), leaving a total of 76 sequenced isolates for genomic analysis.
(PDF)

S2 Fig Maximum likelihood tree for the 76 sequenced isolates based on their concatenated MLST sequences, constructed using MEGA with Tamura-Nei model and 1000 bootstrap replications. Identified *spa* types are listed on the right of each lineage and colored based on host groups (red, community residents; green, household members; blue, hog workers). SLV, single-locus variant with one SNP difference from the closet MLST type; ND, untypable.
(PDF)

S3 Fig Whole-genome inferred phylogeny of the 19 ST5 *S. aureus* isolates sequenced in this study, along with published genome sequences of 5 ST5 strains (ECT-R2, ED98, Mu3, Mu50, N315) and 2 ST105 strains (JH1 and JH9). For each reference strain, the country of report and host (H, human; C, chicken) are listed in parenthesis. Color dots represent host groups in this study (red, community residents; green, household members; blue, hog workers). Asterisks indicate two isolates obtained from the same household. Topology with minor differences from Fig 2 is highlighted by yellow. Isolate genome assemblies were annotated in the CloVR-Microbe pipeline [41]. Annotated genome assemblies were compared in the CloVR-Comparative pipeline [43], which performed whole genome alignment using Mugsy and constructed phylogeny using Phylomark algorithm and FastTree.
(PDF)

S4 Fig **Comparisons of (A) SaPIs and (B) plasmids of the ST5 *S*. *aureus* isolates in this study.** For SaPIs, *attL* and *attR* sites are shown as vertical bars at left and right ends, *int*/*xis* genes shown in green, regulation genes in yellow, phage genes in cyan, virulence factor genes in pink, and ferrichrome-binding protein gene (*fhuD*) and leucine carboxyl methyltransferase gene (*lcm*) in red. Highly identical regions are shown with nucleotide identities. These SaPIs seemed to have evolved from recombination between SaPIs of a ST6 strain (Tokyo12381) [47], a ST239 strain (OC3) [48], and a hospital strain (HHMS2) [49]. For plasmids, heavy metal and antiseptic resistance genes are shown in green, toxin genes (*sed*, *sej*, *ser* and bacteriocin-related ones) in red, and antibiotic resistance genes (*blaZ*, *blaR1*, *blaI*) in pink. Published plasmid sequences with high identities to the plasmids reported in this study are listed in parentheses.
(PDF)

S5 Fig **Venn diagram of lineage diversity of nasal *S*. *aureus* from each population group, based on (A) MLST typing or (B) *spa* typing.** Numbers represent MLST or *spa* types. SLVs are considered as individual MLST types.
(PDF)

S1 Table Published *S. aureus* genomes included in this study. Plasmids are listed if available.
(XLSX)

S2 Table Virulence factors examined in this study. (XLSX)

S3 Table SaPI families examined in this study. (XLSX)

S4 Table Statistics of WGS and *de novo* assembly for the 77 *S. aureus* isolates. Two isolates with low sequencing quality were excluded.
(XLSX)

S5 Table Genomic features of the 76 successfully sequenced *S. aureus* isolates and several published *S. aureus* strains from various hosts. (XLSX)

S6 Table Identified antibiotic resistance genes and their functions. (XLSX)
