# Supplementary figures and images for: Genomic differences between nasal Staphylococcus aureus from hog slaughterhouse workers and their communities
Source: PLoS One. 2018 Mar 6;13(3):e0193820. doi: 10.1371/journal.pone.0193820 (PMC5839586; doi:10.1371/journal.pone.0193820)

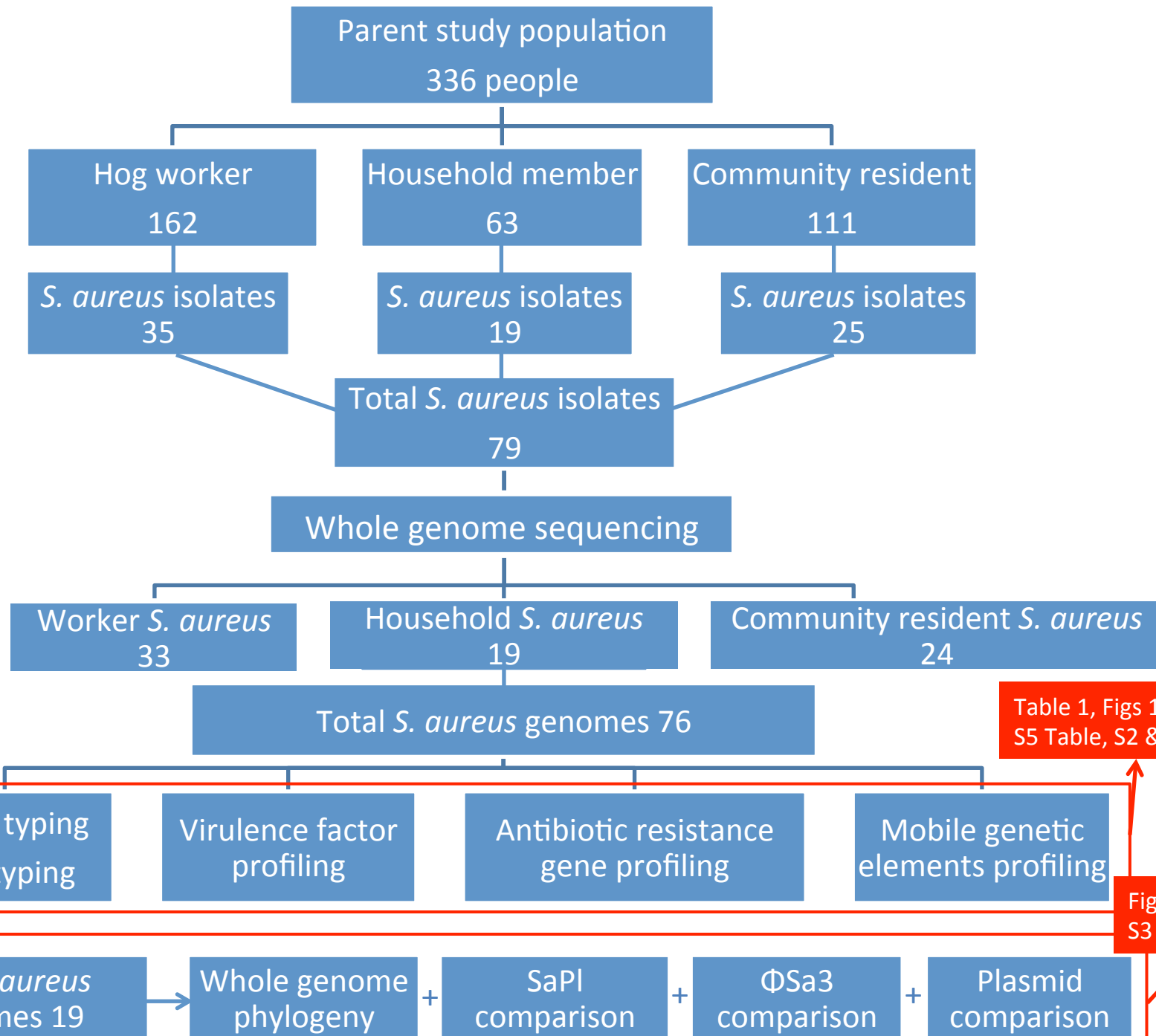

Table 1, Figs 1 & 3  
S5 Table, S2 & S5 Figs

Fig 2,  
S3 & S4 Figs

Supplement: S1 Fig — After WGS, three isolates were excluded due to low sequencing quality (n = 2) or untypable MLST (n = 1), leaving a total of 76 sequenced isolates for genomic analysis. (PDF) [file pone.0193820.s001.pdf]

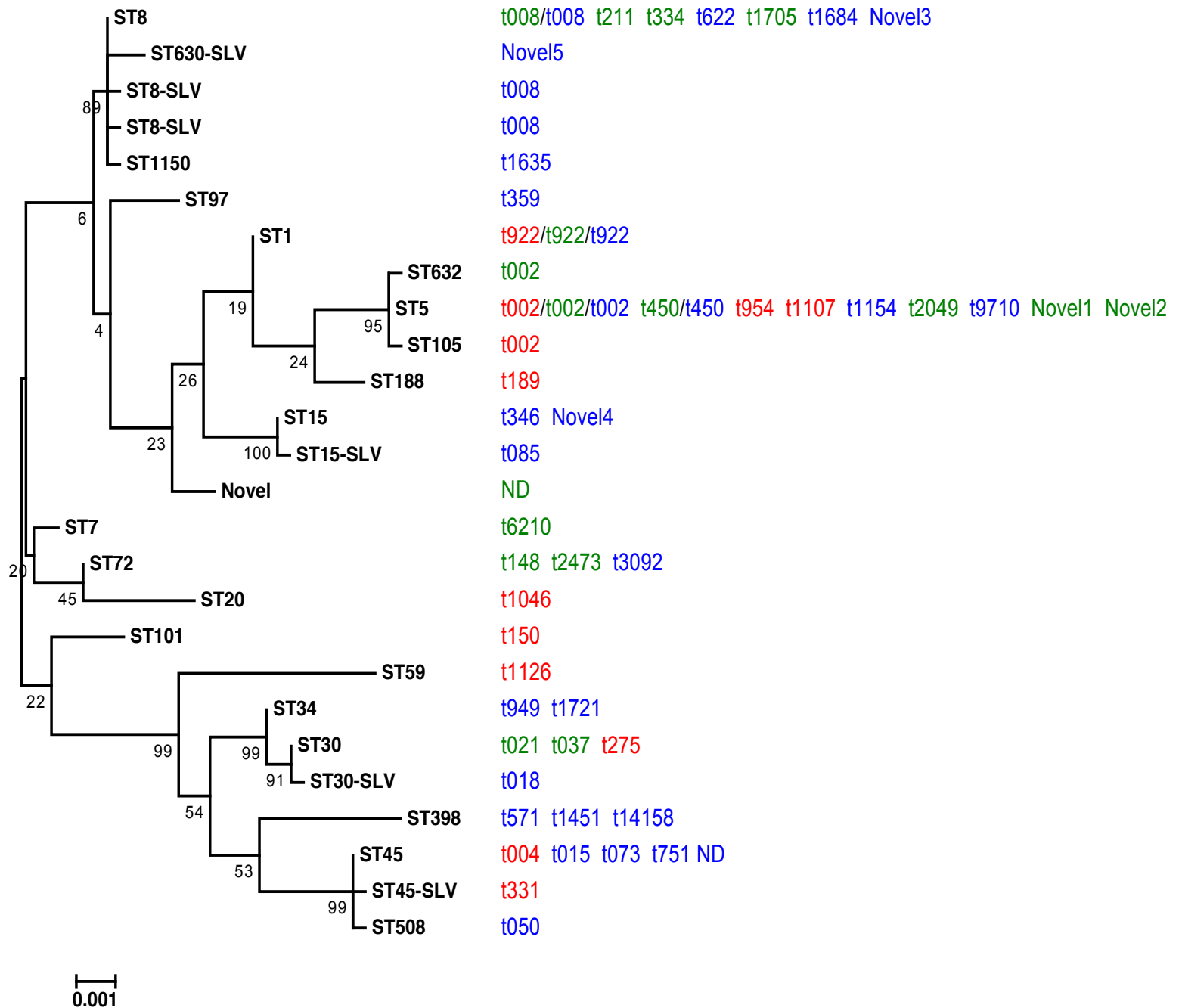

Supplement: S2 Fig — Identified spa types are listed on the right of each lineage and colored based on host groups (red, community residents; green, household members; blue, hog workers). SLV, single-locus variant with one SNP difference from the closet MLST type; ND, untypable. (PDF) [file pone.0193820.s002.pdf]

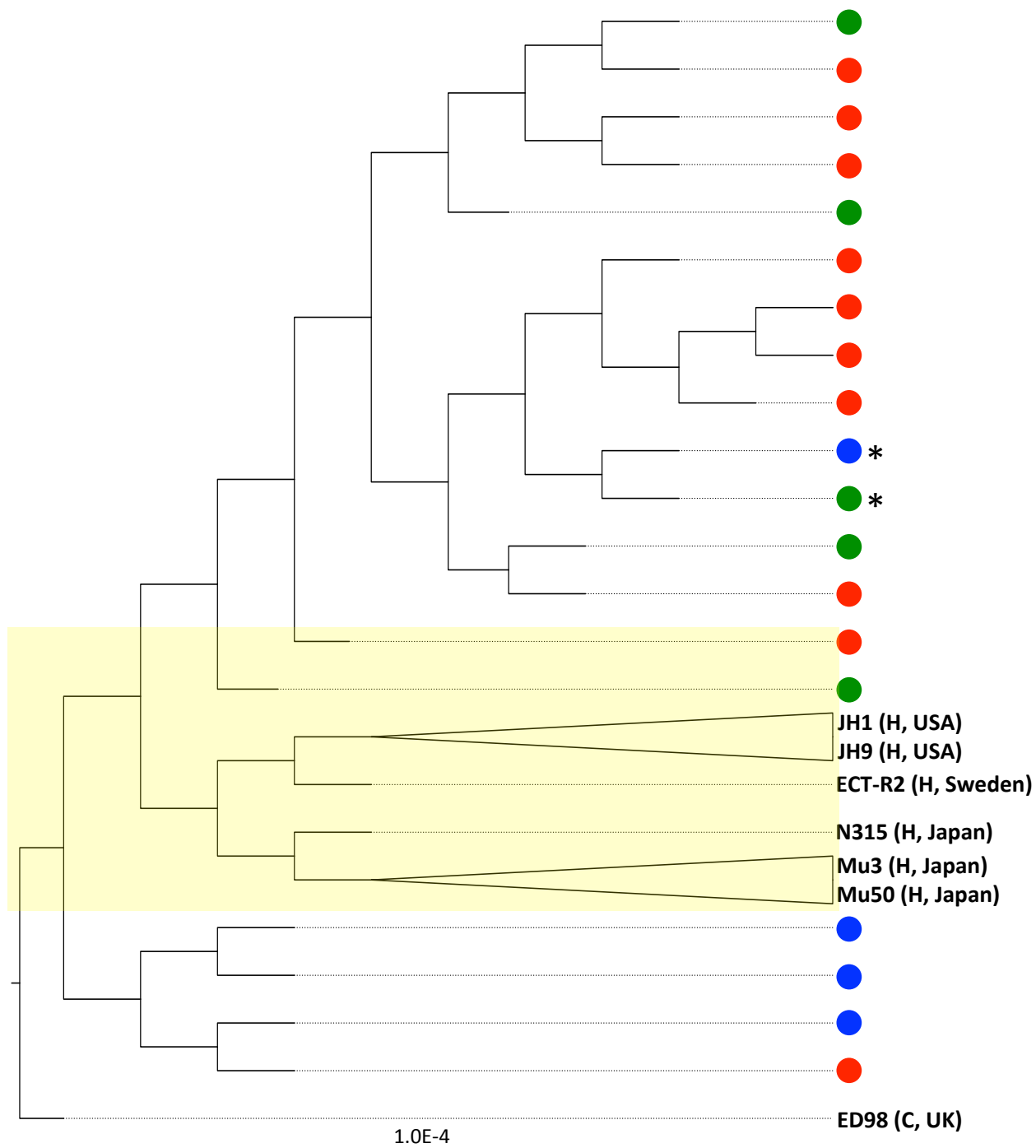

Supplement: S3 Fig — For each reference strain, the country of report and host (H, human; C, chicken) are listed in parenthesis. Color dots represent host groups in this study (red, community residents; green, household members; blue, hog workers). Asterisks indicate two isolates obtained from the same household. Topology with minor differences from Fig 2 is highlighted by yellow. Isolate genome assemblies were annotated in the CloVR-Microbe pipeline [41]. Annotated genome assemblies were compared in the CloVR-Comparative pipeline [43], which performed whole genome alignment using Mugsy and constructed phylogeny using Phylomark algorithm and FastTree. (PDF) [file pone.0193820.s003.pdf]

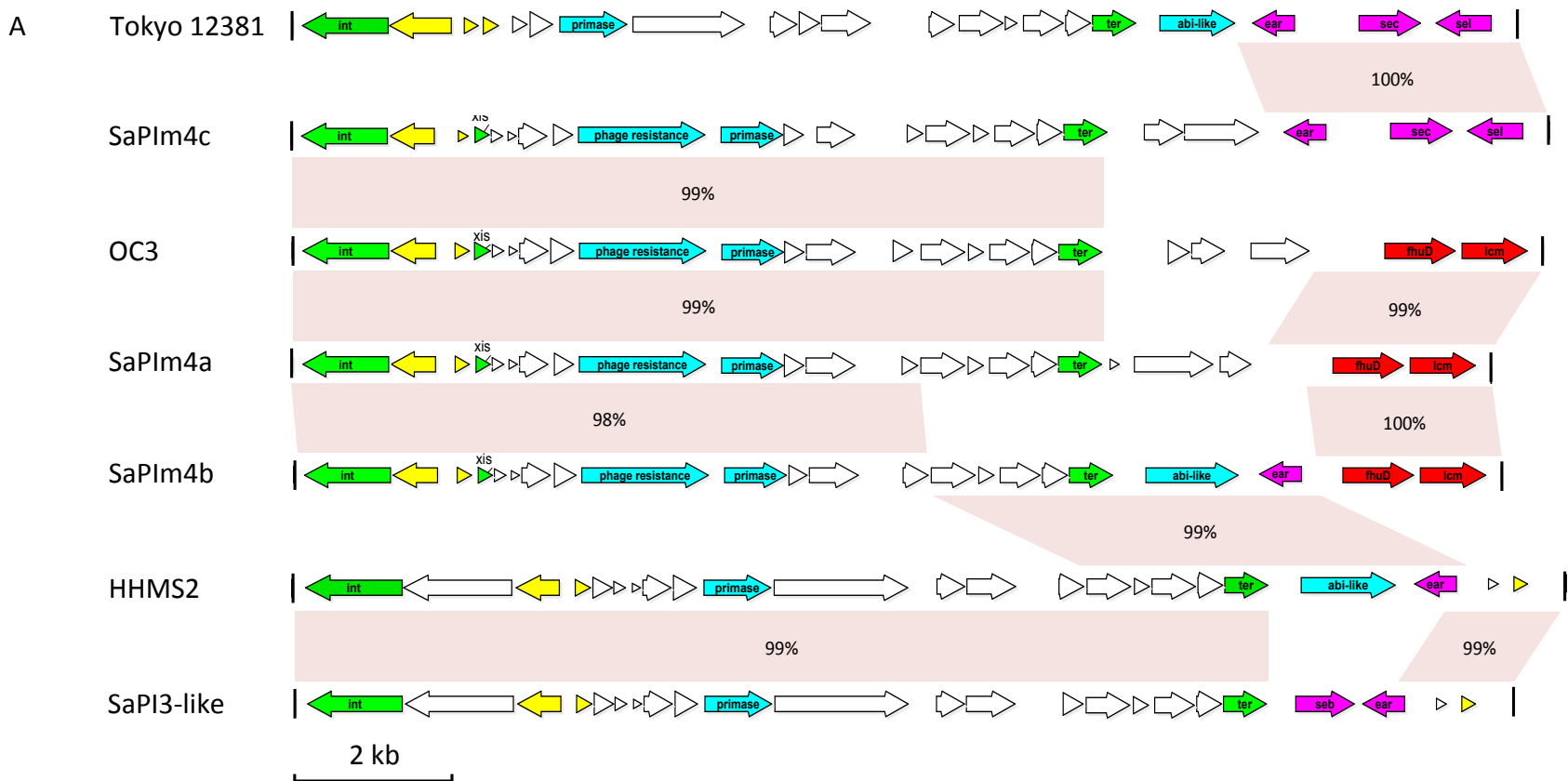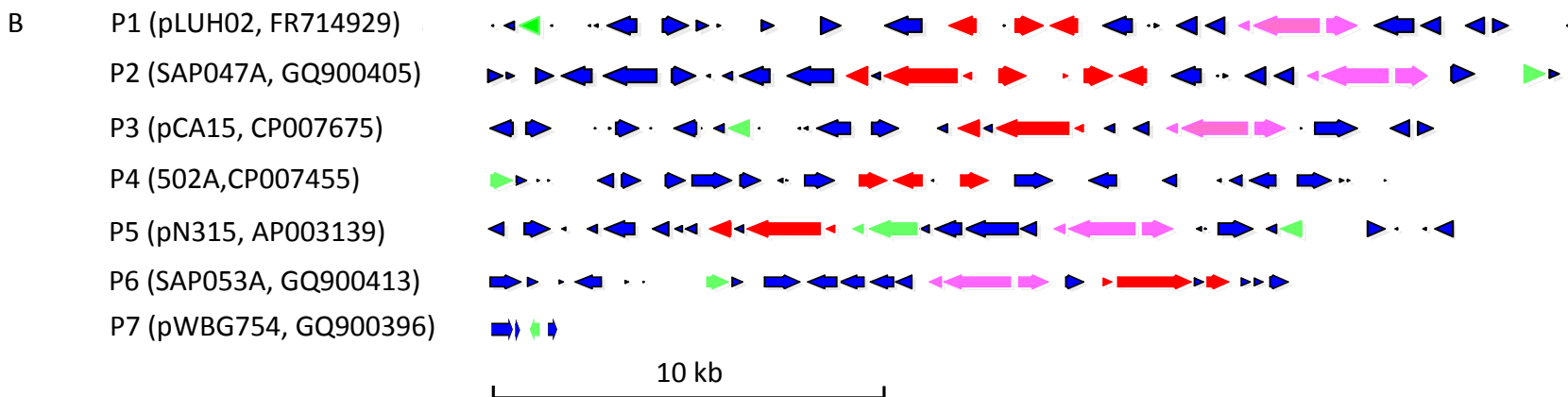

Supplement: S4 Fig — Comparisons of (A) SaPIs and (B) plasmids of the ST5 S. aureus isolates in this study. For SaPIs, attL and attR sites are shown as vertical bars at left and right ends, int/xis genes shown in green, regulation genes in yellow, phage genes in cyan, virulence factor genes in pink, and ferrichrome-binding protein gene (fhuD) and leucine carboxyl methyltransferase gene (lcm) in red. Highly identical regions are shown with nucleotide identities. These SaPIs seemed to have evolved from recombination between SaPIs of a ST6 strain (Tokyo12381) [47], a ST239 strain (OC3) [48], and a hospital strain (HHMS2) [49]. For plasmids, heavy metal and antiseptic resistance genes are shown in green, toxin genes (sed, sej, ser and bacteriocin-related ones) in red, and antibiotic resistance genes (blaZ, blaR1, blaI) in pink. Published plasmid sequences with high identities to the plasmids reported in this study are listed in parentheses. (PDF) [file pone.0193820.s004.pdf]

A

Community residents

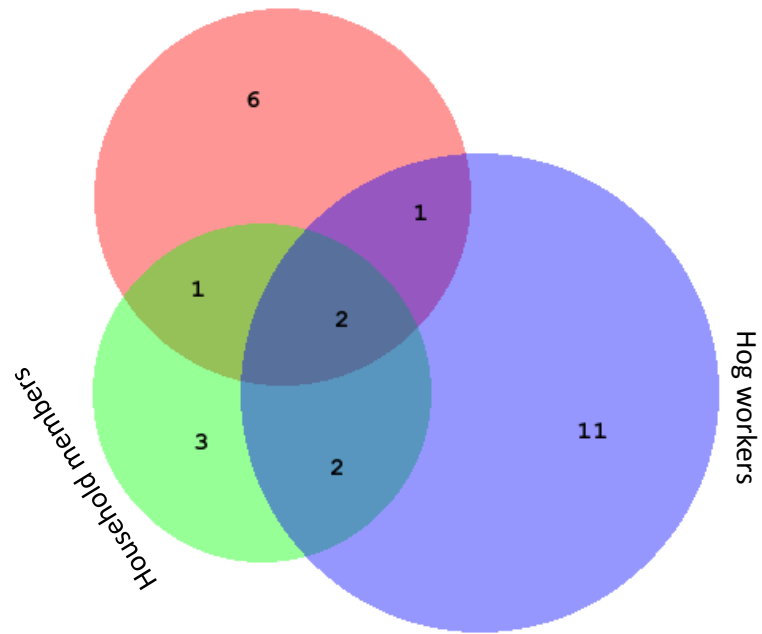

B

Community residents

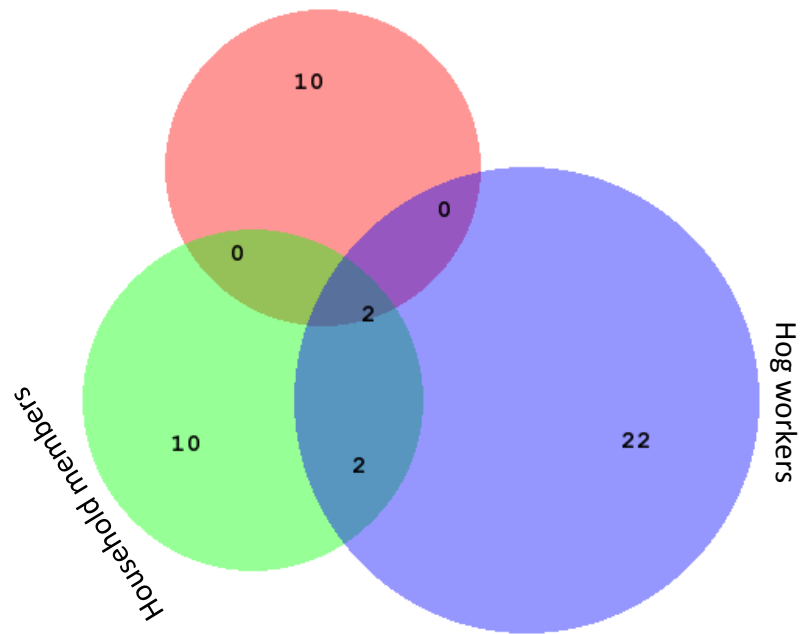

Supplement: S5 Fig — Venn diagram of lineage diversity of nasal S. aureus from each population group, based on (A) MLST typing or (B) spa typing. Numbers represent MLST or spa types. SLVs are considered as individual MLST types. (PDF) [file pone.0193820.s005.pdf]
